# HIV-1 Tat Upregulates the Receptor for Advanced Glycation End Products and Superoxide Dismutase-2 in the Heart of Transgenic Mice

**DOI:** 10.3390/v14102191

**Published:** 2022-10-04

**Authors:** Alaa N. Qrareya, Nason S. Wise, Emmanuel R. Hodges, Fakhri Mahdi, James A. Stewart, Jason J. Paris

**Affiliations:** 1 Department of BioMolecular Sciences, School of Pharmacy, University of Mississippi, Oxford, MS 38677, USA; 2 Research Institute of Pharmaceutical Sciences, University of Mississippi, Oxford, MS 38677, USA

**Keywords:** cardiomyocytes, echocardiography, receptor for advanced glycation end products, superoxide dismutase, trans-activator of transcription

## Abstract

Cardiovascular disorder (CVD) is a common comorbidity in people living with HIV (PLWH). Although the underlying mechanisms are unknown, virotoxic HIV proteins, such as the trans-activator of transcription (Tat), likely contribute to CVD pathogenesis. Tat expression in mouse myocardium has been found to induce cardiac dysfunction and increase markers of endothelial toxicity. However, the role that Tat may play in the development of CVD pathogenesis is unclear. The capacity for Tat to impact cardiac function was assessed using AC16 human cardiomyocyte cells and adult male and female transgenic mice that conditionally expressed Tat [Tat(+)], or did not [Tat(−)]. In AC16 cardiomyocytes, Tat increased intracellular calcium. In Tat(+) mice, Tat expression was detected in both atrial and ventricular heart tissue. Tat(+) mice demonstrated an increased expression of the receptor for advanced glycation end products and superoxide dismutase-2 (SOD-2) in ventricular tissues compared to Tat(−) controls. No changes in SOD-1 or α-smooth muscle actin were observed. Despite Tat-mediated changes at the cellular level, no changes in echocardiographic measures were detected. Tat(+) mice had a greater proportion of ventricular mast cells and collagen; however, doxycycline exposure offset the latter effect. These data suggest that Tat exposure promotes cellular changes that can precede progression to CVD.

## 1. Introduction

Advanced human immunodeficiency virus-1 (HIV-1) treatments have reduced HIV-associated morbidity and mortality [1,2,3]. However, people living with HIV (PLWH) are more likely to develop premature age-related comorbidities compared to age-matched healthy individuals [4,5,6,7,8,9]. As such, PLWH experience cardiovascular diseases (CVDs) such as heart failure [10,11], cardiomyopathy [12], myocardial infarction [13,14], coronary heart disease [15,16,17], and sudden cardiac death [18,19] in greater proportions compared to same-aged seronegative individuals. CVDs are the main leading cause of death globally [20]; however, the risk of CVDs is higher among PLWH [21], and its incidence increases with age [22,23]. The proportion of older PLWH who experience CVDs is expected to further increase (~78%) by 2030 [22]. Antiretroviral therapies cannot entirely eradicate viral proteins or promote full immune system recovery [24,25]. Therefore, persistent viral proteins may contribute to the risk of CVD in HIV.

The trans-activator of transcription (Tat) is a non-structural HIV protein secreted from infected cells, predominantly CD4^+^ T cells, as well as monocytes/macrophages, to enhance viral replication [26,27]. Tat is also found to exert deleterious effects on the myocardium. Tat induces endothelial cell dysfunction, including vasculopathy via the increased expression of vascular cell adhesion molecule-1 [28]; activation of monocyte recruitment and adhesion [29]; and endothelial senescence, mediated in part by microRNA-34 signaling in cultured endothelial cells [30]. Moreover, Tat exerts vascular and atherogenic effects mediated by the NF-κB pathway [29,31,32,33]. In addition to these structural changes, Tat alters mitochondrial function and exacerbates the production of reactive oxygen species (ROS) in cultured cardiomyocytes [34]. Furthermore, Tat induces bradycardia by activating parasympathetic neurons within the nucleus ambiguous [35] and mediates vascular aging in rodents [30]. Although the Tat-mediated mechanisms that may contribute to CVDs are not well understood, Tat has been demonstrated to upregulate the receptor for advanced glycation end products (RAGE) within the blood-brain barrier [36,37,38], a receptor target known to be involved in CVD [39,40].

Advanced glycation end products (AGEs) are non-enzymatic glycation and oxidation products crosslinked to proteins, lipids, and nucleic acids [41]. The accumulation of AGEs increases the risk of CVD by binding to their receptor, RAGE [42]. AGE-RAGE signaling is involved in neointimal hyperplasia formation after vascular injury [43], the exacerbation of ROS production [44,45], the alteration of endothelial function [44], and the attenuation of cardiac fibroblast migration [46]. The formation of endogenous AGEs is accelerated during inflammation, aging, and oxidative stress [41]. As such, levels of circulating AGEs are elevated in PLWH [47,48], concurrent with greater levels of oxidative stress, pro-inflammation [47], and disease-related cardiometabolic biomarkers compared to seronegative people [48]. Furthermore, the levels of soluble RAGE in the serum of PLWH were positively correlated with carotid atherosclerosis [49]. However, the impact of Tat on AGE-RAGE signaling in the myocardium needs to be investigated. Herein, we examined the impact of HIV-1 Tat exposure on the development of cardiovascular abnormalities in adult (2–6 months old) HIV Tat-transgenic mice. We hypothesized that conditional Tat expression would increase the expression of RAGE within the heart concurrent with the cardiac abnormalities.

## 2. Materials and Methods

All experimental procedures were preapproved by the Institutional Animal Care and Use Committee at the University of Mississippi and conducted in accordance with the National Institutes of Health Guide for Care and Use of Laboratory Animals (NIH Publication No. 85-23) ethical guidelines.

### 2.1. Intracellular Calcium Imaging

The human cardiomyocyte AC16 cell line was obtained from Millipore Sigma (#SCC109). Cells were cultured in Dulbecco’s modified Eagle’s medium: nutrient mixture F-12 (DMEM/F-12) containing 2 mM L-glutamine, 12.5% FBS, and 1× penicillin-streptomycin. Cardiomyocytes were seeded (3 × 10^3^ cells/dish) on 35 mm glass-bottom Petri dishes. For calcium measurement, Fluo-4 AM (5 μM) was used and prepared per manufacture instructions (catalog #F10489, Thermo Fisher, Waltham, MA, USA). Cells were loaded with Fluo-4 AM for 30 min prior to Tat treatment. Loaded-cells were incubated at 37 °C for 20 min, then 15 min at room temperature. After incubation, cells were washed twice with Hank’s balanced salt solution. Time-lapse fluorescent microscopy was conducted using an inverted Nikon Ti2E microscope (ex/em: 494 nm/506 nm) over 10 min (1 image/second for the first 2 min; 1 image/30 s for the next 8 min; [50]). Cells were stimulated either with Tat (50 ng/mL: ImmunoDx, Woburn, MA, USA) or water after 30 s of starting live-cell imaging. Images were acquired and analyzed using NIS-analysis software (Nikon, Melville, NY, USA). Four microscopic fields were assessed in each dish. Within each field, fluorescent intensity was assessed for every cell in the field. For each cell, the mean of four randomly selected regions of interest (ROIs) on the soma was measured. Differences in fluorescent intensity from baseline were calculated for each culture using the following formula: f − f_0_/f_0_.

### 2.2. Animal and Housing

Adult (2–6 months old) male (n_Tat(−)_ = 48, n_Tat(+)_ = 63), and female (n_Tat(−)_ = 25, n_Tat(+)_ = 21) HIV-1 Tat transgenic mice were generated in the vivarium at the University of Mississippi (University, MS, USA). Mice were housed (3–5/cage) on a reversed 12:12 h light-dark cycle (lights off at 09:00 h) in a temperature/humidity-controlled environment.

### 2.3. HIV-1 Tat Induction

HIV-1 Tat protein was conditionally expressed via the administration of doxycycline (Dox) hyclate (56 mg/kg; Cayman Chemical, Ann Arbor, MI, USA), as previously described [51]. Briefly, Tat(+) mice express the *tat* transgene and a reverse tetracycline-controlled transactivating (rtTA) transcription factor, whereas Tat(−) mice express only the rtTA transcription factor [51]. In this mouse model, *tat* expression is driven by a GFAP-relegated promoter. While the majority of GFAP^+^ cells reside in the central nervous system [52,53], there is known GFAP expression in the heart, as well [54,55,56].

To induce Tat exposure, Tat(+) and Tat(−) mice received intraperitoneal injections of Dox over 5 consecutive days, followed by 2–21 days of Dox wash-out in order to avoid nonspecific effects of Dox (Dox t_1/2_ = 5–6 h; [57]). As an additional control, some Tat-transgenic mice received saline injections and were included as uninduced negative controls.

### 2.4. Echocardiography

All mice underwent a transthoracic echocardiography protocol using VEVO 3100 Ultrasound system (FUJIFILM VisualSonics, Inc., Toronto, ON, Canada). A 2-dimensional (2D) parasternal short- and long-axis brightness (B-mode) of the mid-left ventricle (LV) was obtained at the level of the papillary muscles. Additionally, a 2D targeted motion-mode (M-mode) tracing was recorded to measure end diastolic and end systolic wall thicknesses and internal chamber dimensions. LV ejection fraction (%EF) and fractional shortening (%FS) were calculated as an index of LV systolic performance. Doppler-based flow velocity measurements were also taken to determine mitral valve E-wave/A-wave (MV E/A) ratios for diastolic LV function. MV E/A ratio marks changes in the peak velocity of blood flow from the LV during early diastole (E-wave) to the peak velocity of blood flow into the LV by atrial contraction during late diastole (A-wave). The MV E/A ratio represents LV filling velocity between systolic periods. Mice were maintained at ~1% isoflurane anesthesia during the echocardiography procedure. A minimum of 5 cardiac cycles were used to generate the data obtained. Heart measurements were conducted as described previously [58,59] after either 2- or 21-days of wash-out from Dox.

### 2.5. Tissue Collection

Following echocardiography measurements, mice were sacrificed via cervical dislocation followed by rapid decapitation and removal of heart. Hearts were collected, weighed, and tissues were flash-frozen on dry ice and stored at −80 °C until later biochemical analysis. Mid-ventricle sections were also fixed in 4% paraformaldehyde for histology.

#### 2.5.1. Cardiac Histology

Paraformaldehyde-fixed ventricular tissues were paraffin-embedded and sectioned (5 μm). Sections were stained with polychromatic methylene blue (mast cells, 600× magnification) or picric acid Sirius red F3BA (PASR, collagen, 200× magnification). Mast cell counts were performed for each of the hearts (n = 6–8/group), and mast cell counts were normalized to scanned images of the stained heart sections. A micrometer was included in each scan to calibrate units of area measurement [60]. Estimates of thick and thin collagen fibrils as a measure of fibrosis in the heart were conducted as previously described [46]. Due to the birefringent nature of the PASR stain, images of collagen fibrils from stained hearts were obtained by passing polarized light collagen, which refracted a distinct color based upon the size of the collagen fibrils: red and yellow (thick filaments) and green (thin filaments). Quantitative analysis was conducted using a light microscopy system (Nikon Eclipse Ti2) with a video-based image-analyzer system. Triphasic analysis color thresholds were set to capture and generate a percent collagen content [(Red (0–40) Green (0–80) Blue (0–255)] per 20× field within the specified RGB wavelength ranges separated from the background [Red (20–255) Green (40–255) Blue (35–255)]. Results are presented as the mean ± SEM values computed from the average of *n* = 25–35 individual measurements obtained from each heart. Cardiac vasculature, epicardium, and endocardium were avoided due to high levels of collagen content that do not accurately reflect myocardial interstitial collagen.

#### 2.5.2. Western Blot

Proteins were isolated from hearts obtained from Tat(−) and Tat(+) mice. Tissues were minced in a Modified Hunter’s Buffer [MHB; 1% Triton X-100,75 mM, NaCl, 5 mM tris pH 7.4, 0.5 mM orthovanadate, 0.5 mM ECTA, 0.5 mM EGTA, 0.25% NP-40, and freshly added Halt Protease Inhibitor Cocktail (100×; Thermo Fisher)]. Minced tissues were incubated on ice with MHB for 10 min, and tissue pieces were then sonicated. Samples were centrifuged for 15 min at 32,000× *g* at 4 °C, and the supernatant was removed and stored at −80 °C. Protein concentrations were assessed using a bicinchoninic acid assay (BCA; Pierce Biotechnology) according to the manufacturer’s instructions. 25 µg of protein per sample was loaded for Western blot analyses. Antibodies used were as follows: monoclonal α-smooth muscle actin (α-SMA; 43 kDa; 1:400; Sigma Aldrich 2547), RAGE (46 kDa; 1:400; Santa Cruz sc-365154), superoxide dismutase-1 (SOD-1; 23 kDa; 1:400; Santa Cruz Biotechnology sc-101523), and superoxide dismutase-2 (SOD-2; 25 kDa; 1:400; Santa Cruz Biotechnology sc-133134). β-tubulin (50 kDa; 1:400; Santa Cruz sc-398937) was used as a loading control. Western blots were visualized using an iBRIGHT imaging system (Thermo Fisher Scientific, Waltham, MA, USA).

Western Immunoblotting for GFAP: 25 μg of heart lysate from Tat(−) and Tat(+) mice was reduced, heated, and run on a 4-20% Tris-glycine SDS-PAGE gel (Bio-Rad Laboratories, Hercules, CA, USA). The gel was run for 1 h at 25’30 mAmp/gel and then transferred to nitrocellulose membrane at 100 volts for 1 h at 4 °C. The membrane was blocked with Odyssey^®^ Blocking Buffer in TBS (Licore, Inc, Lincoln, NJ, USA) for 1 h at room temperature. The membrane was then incubated with primary antibodies to GFAP at 1:1500 dilution (Millipore) or GAPDH at 1:1000 dilution (Santa Cruze) in a blocking buffer with gentle agitation at 4 °C overnight. The membrane was washed with TBST four times for 15 min each time. After washing, the membrane was incubated with an IRDye^®^ 800CW goat anti-mouse against GFAP or IRDye^®^ 680RD donkey anti-goat against GAPDH, both at 1:4000 dilution in blocking buffer for 1 h at room temperature. After three washes with TBST, the electroblotted proteins were detected via a LICOR-Odyssey CLX imager to visualize the protein bands.

#### 2.5.3. RNA Isolation and Quantitative Real-Time Polymerase Chain Reaction

The ventricle and atrium regions of the mouse heart were isolated from each subject and homogenized in TRizol reagent according to manufacture protocol (Thermo Fisher Scientific) followed by Qiagen Clean up kit (Qiagen, Germantown, MD, USA). Total RNA concentration was determined by Nanodrop spectrophotometer (NanoDrop 2000c, Thermo Fisher Scientific). 1 μg of RNA was used and cDNA was made using Revert Aid First Strand cDNA Synthesis (#K1651, Fisher Scientific). All primers were purchased from IDT (Coralville, IA, USA). qRT-PCR reactions were performed to measure Tat mRNA expression using a Bio-Rad CFX Connect Real-Time System (Bio-Rad, Hercules, CA, USA) in 96 well plates (Applied BioSystems, Fisher Scientific). 1 μg of cDNA in a final volume of 25 μL containing 400 nM primers using SYBR Green master mix (Thermo Fisher Scientific) was used for each reaction. The sequence for Tat primers was as follows: forward primer 5′-GCCCTGGAAGCATCCAGGAAGTC-3′, reverse 5′-CGTCGCTGTCTCCGCTTCTTCCT-3′. The sequence for GAPDH primers was as follows: forward 5′-GGAAGCTCACTGGCATGGC-3′, reverse 5′-TAGACGGCAGGTCAGGTCCA-3′. The PCR thermal cycling reaction for each set of primers started with an initial denaturation at 95 °C for 10 min, step cycles at 95 °C for 5 s (denaturation), 58 °C for 10 s (annealing), 72 °C for 20 s (extension) for a total of 40 cycles followed by 72 °C for 5 min. qRT-PCR for the housekeeping gene, GAPDH, was performed in parallel for all reactions. Results are presented as the average of three independently conducted trials.

### 2.6. Statistical Analyses

Data for *tat* mRNA expression and echocardiography were analyzed via two-way analyses of variance (ANOVA). Mast cell counts, collagen content, and Western blot analyses were analyzed via one-way ANOVA. Fluo-4 AM data were analyzed via repeated measures ANOVA using treatment group as the between-subjects factor and time as the within-subjects factor. Group differences following main effects were assessed via Tukey’s honestly significant difference *post hoc* tests. Interactions were delineated via simple main effects and main effect contrasts with α corrected for family wise error. Data were considered significant when *p* ≤ 0.05. All analyses were performed using SAS StatView and the GraphPad Prism software.

## 3. Results

### 3.1. Tat Dysregulated Intracellular Calcium ([Ca^2+^]_i_) in Cultured Human Ventricular Cells

**Figure 1:**
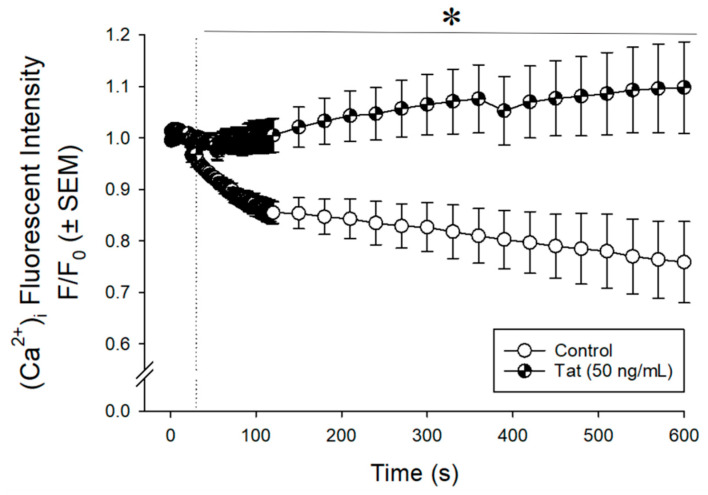
Intracellular Calcium Levels in Cultured Cardiomyocytes. Cells were incubated with Fluo-4 AM and intracellular calcium was measured over 10 min (every second for the first 2 min; and every 30 s for the next 8 min). Tat (50 ng/mL) or vehicle was applied at 30 s. * Indicates Tat-treated cells significantly differed from control at all times post 30 s; *p* ≤ 0.05, repeated measures two-way ANOVA.

We first examined whether Tat exposure would alter Ca^2+^ homeostasis in a cultured AC16 cardiomyocyte cell line. Cultured human ventricular cells exposed to Tat (50 ng/mL; n = 6) demonstrated a significant increase Ca^2+^ influx in a time-dependent manner compared to vehicle treatment (n = 3; F _(135,945)_ = 5.95, *p* < 0.05; see * Figure 1). In Tat-treated cultures, all time-points following the addition of Tat at 30 s demonstrated a significant increase in [Ca^2+^]_i_ compared to control-treated cultures.

### 3.2. Tat Induction Increased Tat mRNA Levels in the Whole Heart among Adult Male Mice

**Figure 2:**
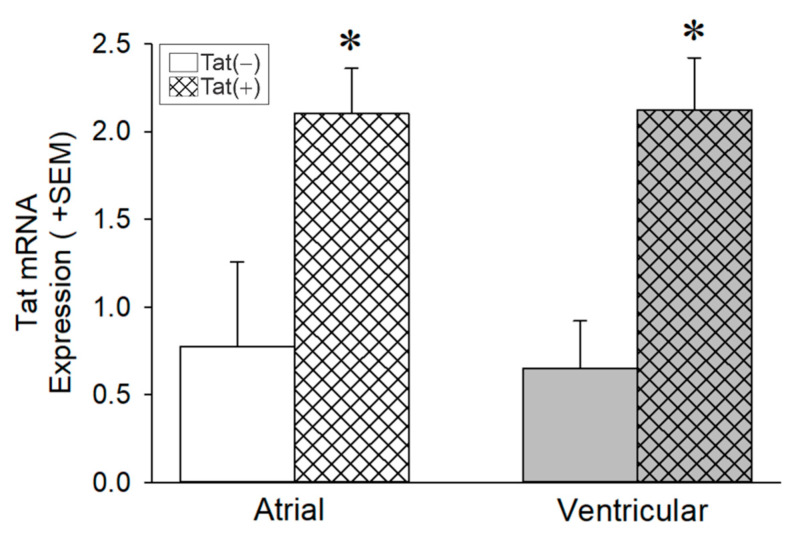
*tat* mRNA Expression in the Heart. *tat* mRNA expression was detected in the whole heart of Tat(+) mice (hatched bars) compared to their control Tat(−) mice (open bars; n = 2–3 per group). * indicates a main effect of genotype wherein Tat(+) mice differ from Tat(−) controls; *p* < 0.05; two-way ANOVA.

We assessed the effect of conditional HIV-1 Tat induction on the presence of *tat* transgene within the heart of adult male mice via qRT-PCR. Transgenic HIV-1 Tat mice, (n_Tat(−)_ = 2, n_Tat(+)_ = 3) were injected with Dox (56 mg/kg) for 5 days with 2 days of washout. There was a main effect of Tat genotype, wherein Tat(+) mice had greater *tat* mRNA expression in either the atrial or ventricular heart chambers compared to control Tat(−) mice (F _(1,6)_ = 18.27, *p* ≤ 0.05; see * Figure 2). We did not observe differences in Tat expression between heart chambers, atrial and ventricular. Given that Tat protein expression is driven by GFAP in this mouse model, we did confirm the expression of GFAP protein expression within the heart of Tat(−) and Tat(+) mice (Figure S1). Thus, *tat* mRNA expression was detected in the whole heart of adult mice.

### 3.3. Tat Induction Did Not Alter Cardiac Function among Adult Mice

**Figure 3:**
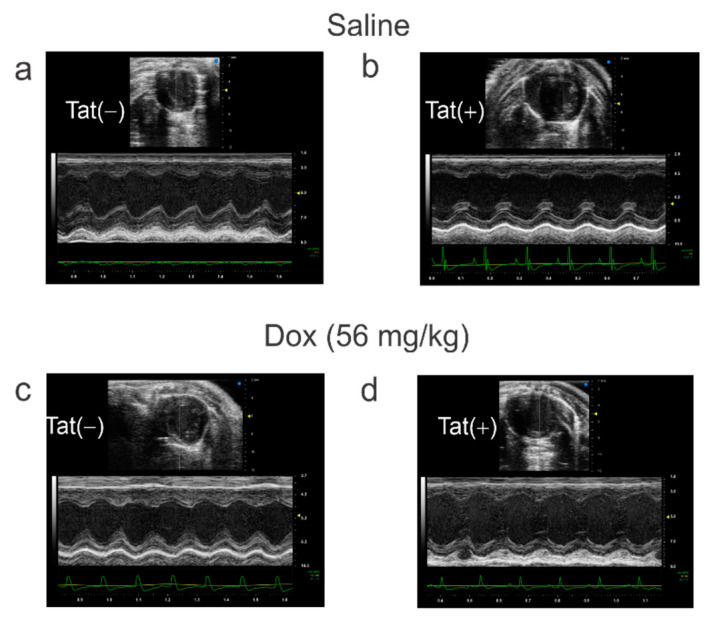
Two-Dimensional Echocardiographic Images Measured by Ultrasound. Tat expression did not alter cardiac function among adult Tat-transgenic mice [Tat(+)] compared to their non-Tat-expressing age-matched counterparts [Tat(−)]. (**a**) Tat(−) mice treated with saline, (**b**) Tat(+) mice treated with saline, (**c**) Tat(−) mice treated with Dox, (**d**) Tat(+) mice treated with Dox.

Given the acute changes observed for Tat to increase [Ca^2+^]_i_, we next investigated whether Tat expression in the heart would interfere with myocardium function using Tat-transgenic male and female adult mice. Neither Tat genotype nor exposure to Dox significantly influenced cardiac physiological function when assessed by B-mode or M-mode using ultrasound (Table 1). The heart rates and the proportion of ejection fraction remained unchanged across all groups (Table 1). We did not observe any alteration of cardiac function between saline (Table 1, Figure 3a,b) or Dox-treated (56 mg/kg) mice (Table 1, Figure 3c,d).

### 3.4. Tat Induction Upregulated RAGE and SOD-2 in Ventricular Tissue of Adult Mice

**Figure 4:**
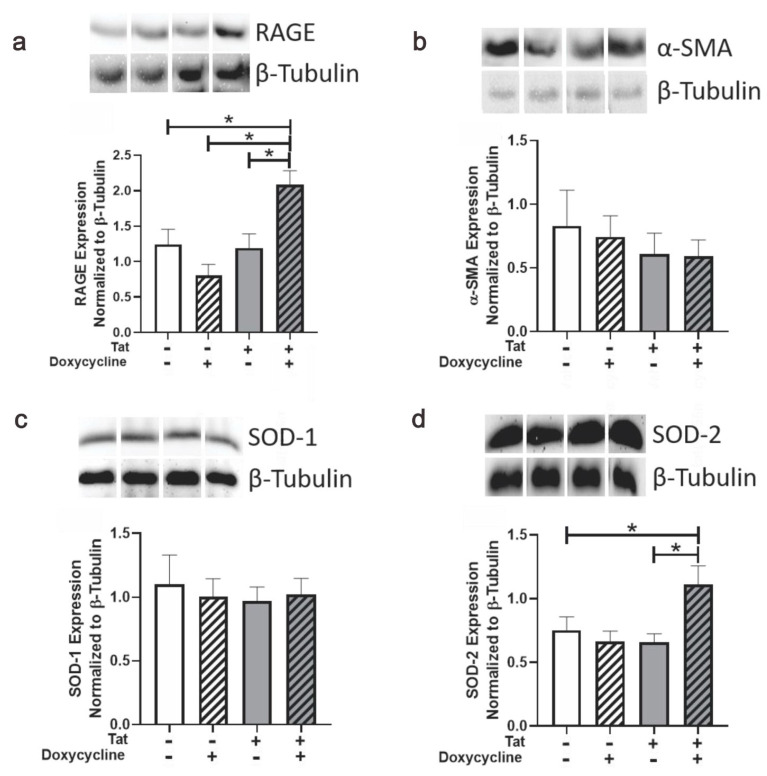
Effect of Tat Expression on the Cardiac Biomarkers of Transgenic Tat Adult Male Mice. Compared to Tat(−) controls, Tat(+) mice (n = 7−8/group) demonstrated upregulation of (**a**) RAGE and (**d**) SOD-2, but not (**b**) α−SMA or (**c**) SOD-1; * *p* ≤ 0.05; one-way ANOVA followed by Tukey’s HSD.

To further investigate the effect of Tat exposure on the cardiac function, we examined the levels of RAGE, α-SMA, and antioxidant proteins (SOD-1 and SOD-2) within the hearts of male Tat-transgenic mice. Inducing Tat with Dox significantly increased RAGE expression in the heart [*F*_(3, 17)_ = 7.426 *p* ≤ 0.05; see *, Figure 4a]. Neither Tat nor treatment with Dox influenced expression of α-SMA (Figure 4 b) or the cytosolic antioxidant copper/zinc-SOD-1 (Figure 4c). However, Tat expression induced by Dox significantly upregulated the mitochondrial antioxidant magnesium-SOD-2 [*F*_(3, 18)_ = 4.359, *p* ≤ 0.05; see * Figure 4d). Dox alone did not account for changes in the expression of any analyte assessed, given that Dox-treated Tat(−) mice did not differ from saline-treated Tat(−) mice on any measure (Figure 4a–d). Thus, Tat induction significantly influenced the expression of RAGE and mitochondrial antioxidant SOD-2 in adult mice despite exerting no functional changes within the hearts.

### 3.5. Tat Exposure Altered Mast Cell Numbers and Collagen Deposition within Ventricular Tissue of Mice

**Figure 5:**
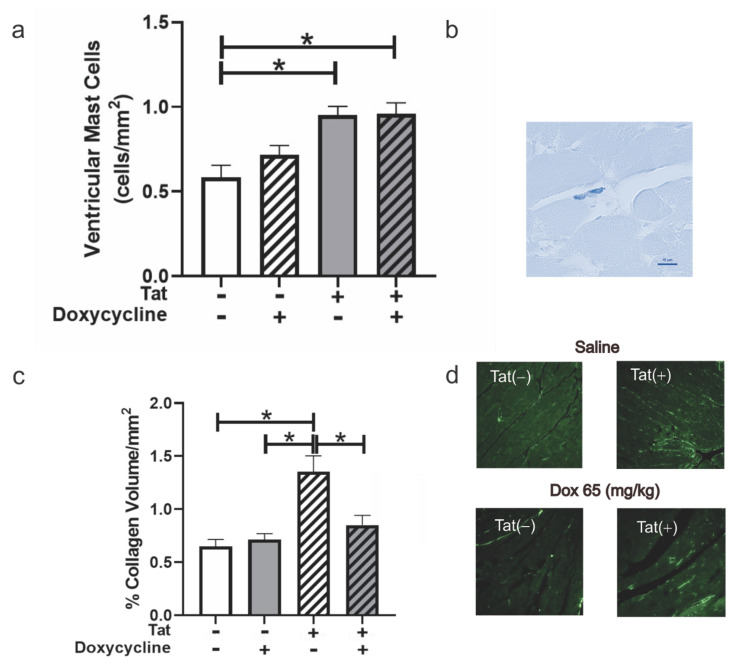
Mast Cells and Collagen Deposition in the Heart of Tat-Transgenic Adult Male Mice or their non-Tat-expressing counterparts. Tat expression influenced pro-inflammatory mast cell releases and extracellular collagen deposition in Tat(+) mice (n = 7–8/group). (**a**) Mast cell numbers and (**b**) a representative photomicrograph of stained mast cells. (**c**) Proportional collagen deposition and (**d**) PASR-stained representative images; * *p* ≤ 0.05; one-way ANOVA followed by Tukey’s HSD. Scale bar = 10 µm.

We also assessed the effect of Tat induction on the cardiac extracellular matrix. Unexpectedly, the mast cell population was upregulated in Tat(+) mice, irrespective of DOX exposure [*F*(_3,22_) = 8.971, *p* < 0.05; see *, Figure 5a,b). Similarly, collagen deposition was increased in the ventricular tissue of Tat(+) mice administered saline, but not those administered Dox, which may have exerted an ameliorating effect [*F*(_3,24_) = 9.958, *p* < 0.05; see * Figure 5c,d)].

## 4. Discussion

In the era of antiretroviral therapy, CVDs occur prematurely and independently of traditional risk factors among PLWH [61,62,63]. The underlying mechanisms by which HIV contributes to CVDs are multifactorial [64,65,66,67,68]. However, it is unknown how viral proteins, including Tat, contribute to CVDs. This study is the first, to our knowledge, to investigate the effect of GFAP-driven Tat expression on the pathogenesis of cardiac dysfunction. In cultured human cardiomyocytes, Tat increased [Ca^2+^]_i_ demonstrating its capacity to exert acute effects on ion flux in heart cells. These findings have been demonstrated to be responsible for changes in cardiac contractility and dysfunction [69,70]. Although the *tat* transgene was detected in the atrium and ventricular chambers of the heart in Tat(+) mice, we did not observe an overall significant change in cardiac function. However, earlier biochemical changes in the heart indicative of cardiac dysfunction were detected. Tat(+) mice demonstrated an increased expression of RAGE and the mitochondrial antioxidant, SOD-2. Prior studies have established an association between SOD-2 upregulation and mitochondrial dysfunction [71,72], resulting in cardiac abnormalities [73,74,75]. Together, these findings support the notion that Tat exposure in adult mice promotes changes in cardiac protein markers consistent with mitochondrial dysfunction. These events may occur prior to cardiac structural and/or functional changes. Thus, these findings suggest that Tat is sufficient to induce changes in the myocardium of adult HIV-1 Tat-expressing mice.

Echocardiographic abnormalities characterized by functional and structural changes are commonly reported among young adults living with HIV [11,74]. Therefore, PLWH are more vulnerable to develop cardiac dysfunction [75,76,77,78,79] and LV hypertrophy [75,78,79]. Independent of HIV, several underlying factors are associated with diastolic dysfunction, and metabolic disorders [75,80,81,82,83]. In this work, we demonstrated that Tat exposure did not significantly alter diastolic or systolic function in Tat-transgenic mice at a young adult age. Our data are consistent with prior preclinical findings using Tat-exposed young mice that reported no functional change in heart physiology [84,85]. However, LV dysfunction was observed at 6 months of age among transgenic mice that expressed Tat in the myocardium, concurrent with diastolic and systolic dysfunction [84,85]. These findings support the notion that cardiac biomarkers associated with Tat-mediated abnormalities may appear at younger ages and precede ventricular dysfunction and heart remodeling/failure. Further studies are required to assess these changes in an older cohort of mice. As well, greater investigation of the potential Tat-mediated mechanisms responsible for CVDs, including the alteration of mitochondrial function and Ca^2+^ homeostasis, should be explored.

Mitochondrial function plays a vital role in maintaining cardiac function and the buffering of Ca^2+^ [86,87,88]. Alterations in mitochondrial structure and/or function contribute to numerous cardiomyopathies [34,71,86]. Studies have demonstrated that Tat-induced mitochondrial dysfunction occurs in cultured neurons [89,90]. Similarly, Tat effects on mitochondrial function can also occur in the myocardium. In the Tat-targeted myocardial model, the overexpression of Tat was associated with mitochondrial damage, including swelling, cristae and matrix disruption, and incomplete fusion [84,86]. Tat also impaired mitochondrial quality control, reduced bioenergetic capacity, disrupted mitochondrial membrane potential, and amplified ROS formation in cultured cardiomyocytes [34]. We and others have found that Tat upregulates [Ca^2+^]_i_ in cultured cardiomyocytes, which is expected to increase the risk of CVDs [34,91,92]. Although the underlying mechanisms of Tat-induced mitochondrial injury are not fully understood, changes in redox status might contribute to Tat-induced mitochondrial dysfunction in cultured cardiomyocytes and astrocytes. However, pretreatment with superoxide dismutase attenuated Tat’s effect on astrocytes [93]. Manganese SOD-2 is an antioxidant-protecting protein localized solely in the mitochondria (mitochondrial matrix and intermembrane space; [94,95,96]). SOD-2 deficiency contributes to cardiac mitochondrial injury, dilated heart chambers [97], a higher incidence of ventricular remodeling, and cardiac fibrosis [73,74]. A higher SOD-2 level was reported in Alzheimer’s disease, which would be considered a compensatory response to attenuate oxidative stress damage [98,99]. Interactions with additional virotoxic HIV proteins may also contribute to CVDs. In support, the level and activity of SOD-2 were augmented in cultured astrocytes in response to the HIV envelope protein, gp120 [100]. In this study, we find increased SOD-2 expression, which may be a compensatory mechanism in response to Tat-induced mitochondrial dysfunction and the failure of Ca^2+^ handling in cardiomyocytes. In addition to Tat-mediated mitochondrial injury, we demonstrated increased mast cell numbers in the cardiac tissue. Mast cell-derived proinflammatory substances such as histamines, cytokines, leukotrienes, and prostaglandins promote cardiac fibrosis during long-term heart remodeling [100,101,102]. Thus, these findings suggest the association between immune dysregulation and CVD in HIV. In support of this, inmate immune activation, including activated monocytes and cardiac macrophages, contributes to CVDs [103,104]. Accordingly, elevated SOD-2 levels might attenuate Tat-mediated mast cell activation at younger ages. However, the impact of prolonged mast cell activation is not well understood. Together, the capacity of Tat to induce cardiomyopathies via mitochondrial injury, overload of Ca^2+^, and increased mast cell number may be an important contributor to cardiac dysfunction in HIV.

RAGE also plays an important role in the pathology of several age-related comorbidities, such as CVDs [104], Alzheimer’s disease [105], and osteoarthritis [106]. RAGE has been demonstrated to be upregulated with aging and accumulates mainly within the heart [107]. Accordingly, RAGE is also involved in mitochondrial dysfunction, including impaired mitochondrial biogenesis, enhanced oxidative stress, altered mitochondrial membrane potential, increased mtDNA damage, and induced mitochondrial death [108,109,110,111]. In addition to mitochondrial damage, RAGE contributes to aortic structural changes characterized by increased collagen deposition in rodents [108,112]. Recent studies have indicated that HIV-1 Tat-induced RAGE expression within the blood–brain barrier accelerates amyloid beta deposition [36,37,38]. The influence of RAGE within the heart could also be an underlying mechanism for Tat-induced CVDs. Consistent with prior findings, we observed Tat exposure to significantly increased RAGE expression in the hearts of young adult male mice, which might be an early biomarker for increased CVD risk. Inhibiting RAGE signaling might serve as a potential therapeutic intervention in PLWH to lower the risk of premature cardiovascular disorders.

The present data must be considered in relation to some caveats. Mast cell number and collagen deposition increased in Tat(+) mice irrespective of Dox treatment. Given that Dox exerts anti-inflammatory effects on its own, its administration may have masked some effects. In support, mast cells were elevated in Tat(+) mice administered saline which could be due to ‘leaky’ expression of Tat in this model [113], but this was not further elevated by Dox administration. Likewise, collagen deposition increased in uninduced Tat(+) mice, Dox administration completely abrogated this effect. The temporal nature of Tat induction by Dox may also play a role in these surprising effects. As such, these findings should also be replicated in Dox-independent animal models.

## 5. Conclusions

These data demonstrate that GFAP-driven Tat expression promotes cardiac abnormalities among adult mice. In support, we determined *tat* mRNA expression in the whole heart concurrent with the dysregulation of calcium homeostasis, the upregulation of RAGE, mitochondrial antioxidant SOD-2, and the mast cell-derived inflammatory response. However, we observed no changes in cardiac functions at young ages. Therefore, these alterations within the myocardium may serve as early biomarkers of CVD before developing cardiac dysfunction among PLWH.

## Figures and Tables

**Table 1:** Morphological and cardiac functional assessments by echocardiogram parameters including brightness- (B-mode) and motion-mode (M-mode) measurements among HIV-1 Tat transgenic mice treated with saline or Dox.

Gender	**Male**				**Female**			
Tat Genotype	**Tat(−)**	**Tat(+)**	**Tat(−)**	**Tat(+)**	**Tat(−)**	**Tat(+)**	**Tat(−)**	**Tat(+)**
n-value	8	13	5	15	11	12	24	22
Treatment	**Saline**		**Dox 56 mg/kg**		**Saline**		**Dox 56 mg/kg**	
HR (bpm)	454.09 ± 49.21	488.37 ± 52.74	473.52 ± 45.52	477.33 ± 70.09	465.07 ± 88.05	465.07 ± 88.05	470.70 ± 53.07	483.51 ± 98.17
**B-mode**					**B-mode**			
SV (µL)	18.21 ± 3.44	24.38 ± 4.82	20.04 ± 7.98	19.83 ± 5.45	17.69 ± 4.13	17.29 ± 3.24	19.75 ± 6.26	20.20 ± 5.63
%EF	48.66 ± 5.61	50.33 ± 9.22	49.71 ± 8.49	47.49 ± 9.67	44.52 ± 9.78	53.31 ± 11.05	52.02 ± 9.22	55.28 ± 7.76
%FS	15.10 ± 4.44	14.01 ± 5.81	13.11 ± 5.92	12.18 ± 4.20	10.11 ± 3.98	12.69 ± 4.22	13.45 ± 5.18	13.52 ± 5.76
CO (mL/min)	8.02 ± 1.59	11.73 ± 1.73	9.14 ± 2.77	9.40 ± 3.07	8.23 ± 2.70	8.31 ± 2.67	9.16 ± 2.77	9.89 ± 3.68
Area (mm^2^)	11.91 ± 3.67	14.47 ± 3.70	13.84 ± 5.62	12.75 ± 3.51	14.45 ± 4.01	9.98 ± 2.13	10.99 ± 2.00	11.73 ± 2.73
Area, s (mm^2^)	11.10 ± 2.32	13.39 ± 3.24	12.18 ± 4.27	12.33 ± 2.69	12.66 ± 2.35	9.66 ± 1.75	10.68 ± 1.82	10.15 ± 2.05
Area, d (mm^2^)	16.73 ± 2.54	20.25 ± 2.86	17.61 ± 5.13	18.16 ± 3.07	17.91 ± 2.48	15.20 ± 1.31	16.81 ± 2.62	16.39 ± 2.51
Volume (µL)	24.10 ± 12.14	30.55 + 11.56	29.94 ± 17.20	24.25 ± 10.25	30.18 ± 13.00	17.29 ± 6.68	18.38 ± 5.05	22.25 ± 9.68
Volume, s (µL)	20.41 ± 7.06	26.70 ± 10.18	23.74 ± 11.05	22.58 ± 7.45	23.48 ± 6.73	16.03 ± 5.51	17.48 ± 4.38	16.60 ± 5.12
Volume, d (µL)	38.62 ± 9.94	51.07 ± 11.39	20.04 ± 7.98	42.41 ± 10.55	41.17 ± 8.66	33.31 ± 5.44	37.24 ± 8.27	36.79 ± 9.44
**M-mode**					**M-mode**			
%EF	63.15 ± 14.73	55.27 ± 16.51	55.42 ± 6.98	62.91 ± 10.61	61.30 ± 6.39	61.77 ± 11.27	63.48 ± 7.18	64.55 ± 6.62
%FS	34.77 ± 10.05	29.97 ± 11.94	28.46 ± 4.31	33.85 ± 7.51	32.28 ± 4.49	33.21 ± 7.07	33.70 ± 5.26	34.45 ± 4.78
IVS, d (mm)	1.08 ± 0.19	0.99 ± 0.23	0.75 ± 0.05	1.16 ± 0.26	0.89 ± 0.21	1.04 ± 0.24	1.13 ± 0.24	0.91 ± 0.20
IVS, s (mm)	1.48 ± 0.21	1.38 ± 0.38	1.07 ± 0.06	1.50 ± 0.28	1.30 ± 0.23	1.37 ± 0.21	1.56 ± 0.25	1.35 ± 0.25
LVID, d (mm)	3.27 ± 0.36	3.65 ± 0.59	3.65 ± 0.32	3.28 ± 0.39	3.36 ± 0.36	3.07 ± 0.30	3.09 ± 0.37	3.12 ± 0.37
LVID, s (mm)	2.15 ± 0.49	2.64 ± 0.78	2.62 ± 0.31	2.19 ± 0.38	2.28 ± 0.31	2.04 ± 0.27	2.06 ± 0.32	2.04 ± 0.32
LVPW, d (mm)	1.22 ± 0.27	1.01 ± 0.23	0.91 ± 0.16	1.08 ± 0.20	0.97 ± 0.31	1.02 ± 0.20	1.16 ± 0.31	1.09 ± 0.28
LVPW, s (mm)	1.57 ± 0.33	1.33 ± 0.25	1.29 ± 0.20	1.43 ± 0.23	1.30 ± 0.27	1.37 ± 0.20	1.46 ± 0.28	1.43 ± 0.25

HR: heart rate; SV: stroke volume; EF: ejection fraction; FS: fractional shortening; CO: cardiac output; s: systolic; D: diastolic; IVS: interventricular septum; LVID: left ventricle inner dimension; LVPW: left ventricular posterior wall; Dox: doxycycline.

## Data Availability

Data available upon request.

## Supplementary Materials

The following supporting information can be downloaded at: https://www.mdpi.com/article/10.3390/v14102191/s1, Figure S1: Expression of GFAP in the heart of Tat-transgenic mice.
