# Supplementary material for: HIV-1 Tat Upregulates the Receptor for Advanced Glycation End Products and Superoxide Dismutase-2 in the Heart of Transgenic Mice
Source: Viruses. 2022 Oct 4;14(10):2191. doi: 10.3390/v14102191 (PMC9607872; doi:10.3390/v14102191)
Supplement: Supplementary file 1 [file viruses-14-02191-s001.zip › viruses-1899761-supplementary.pdf]

Supplementary Materials

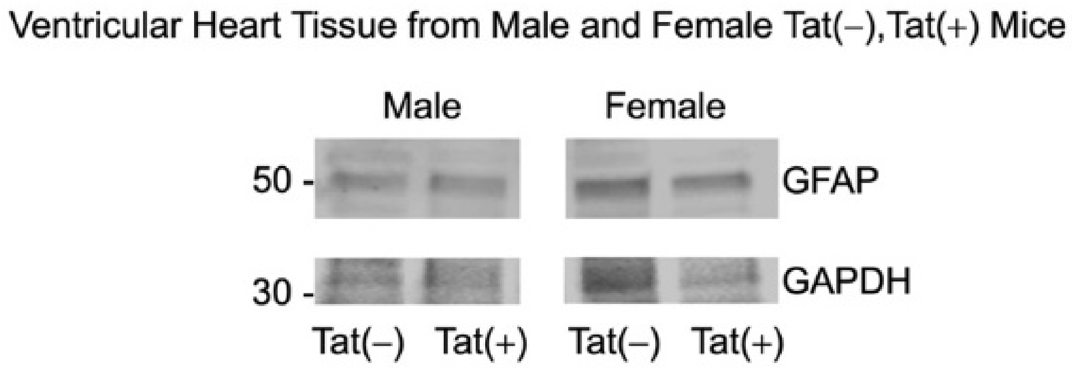

Figure S1. Expression of GFAP in the heart of Tat-transgenic mice. The expression of GFAP within ventricular heart tissue of Tat(–) and Tat(+) mice was confirmed.

Method and Material

Western Immunoblotting for GFAP: 25 μg of heart lysate from Tat(–) and Tat(+) mice was reduced, heated, and run on a 4-20% Tris-glycine SDS-PAGE gel (Bio-Rad laboratories, Hercules, CA). The gel was run for 1 h at 25-30 mAmp/gel and then transferred to nitrocellulose membrane at 100 volts for 1 h at 4°C. The membrane was blocked with Odyssey® Blocking Buffer in TBS (Licore, Inc, Lincoln, NJ) for 1 h at room temperature. The membrane was then incubated with primary antibodies to GFAP at 1:1500 dilution (Millipore) or GAPDH at 1:1000 dilution (Santa Cruze) in blocking buffer with gentle agitation at 4°C for overnight. The membrane was washed with TBST four times for 15 minutes each time. After washing, the membrane was incubated with an IRDye® 800CW goat anti-mouse against GFAP or IRDye® 680RD donkey anti-goat against GAPDH, both at 1:4000 dilution in blocking buffer for 1 h at room temperature. After three washes with TBST, the electroblotted proteins were detected via a LICOR-Odyssey CLX imager to visualize the protein bands.
